# Possible Roles for Basal Levels of (p)ppGpp: Growth Efficiency Vs. Surviving Stress

**DOI:** 10.3389/fmicb.2020.592718

**Published:** 2020-10-09

**Authors:** Llorenç Fernández-Coll, Michael Cashel

**Affiliations:** Intramural Research Program, Eunice Kennedy Shriver NICHD, NIH, Bethesda, MD, United States

**Keywords:** (p)ppGpp, balanced growth, overt starvation, basal levels, destabilized stable RNA

## Abstract

Two (p)ppGpp nucleotide analogs, sometimes abbreviated simply as ppGpp, are widespread in bacteria and plants. Their name alarmone reflects a view of their function as intracellular hormone-like protective alarms that can increase a 100-fold when sensing any of an array of physical or nutritional dangers, such as abrupt starvation, that trigger lifesaving adjustments of global gene expression and physiology. The diversity of mechanisms for stress-specific adjustments of this sort is large and further compounded by almost infinite microbial diversity. The central question raised by this review is whether the small basal levels of (p)ppGpp functioning during balanced growth serve very different roles than alarmone-like functions. Recent discoveries that abrupt amino acid starvation of *Escherichia coli*, accompanied by very high levels of ppGpp, occasion surprising instabilities of transfer RNA (tRNA), ribosomal RNA (rRNA), and ribosomes raises new questions. Is this destabilization, a mode of regulation linearly related to (p)ppGpp over the entire continuum of (p)ppGpp levels, including balanced growth? Are regulatory mechanisms exerted by basal (p)ppGpp levels fundamentally different than for high levels? There is evidence from studies of other organisms suggesting special regulatory features of basal levels compared to burst of (p)ppGpp. Those differences seem to be important even during bacterial infection, suggesting that unbalancing the basal levels of (p)ppGpp may become a future antibacterial treatment. A simile for this possible functional duality is that (p)ppGpp acts like a car’s brake, able to stop to avoid crashes as well as to slow down to drive safely.

## (p)ppGpp, Many Ways to Transfer a Pyrophosphate Group Back and Forth

Synthetases of (p)ppGpp transfer the intact 5'-βγ pyrophosphate group from ATP to the ribose 3' hydroxyl group of GTP or GDP, while (p)ppGpp hydrolases regenerate the GDP and GTP substrates by removal of the same pyrophosphate (Cashel and Kalbacher, 1970; Que et al., 1973; Sy and Lipmann, 1973; Kari et al., 1977). Many combinations of synthetase and hydrolase proteins are found throughout bacterial and plant kingdoms, along with uncertainty of functional implications or even the identities of the alarmone produced (Atkinson et al., 2011; Avilan et al., 2019; Ronneau and Hallez, 2019; Sobala et al., 2019; Jimmy et al., 2020).

No synthetases of (p)ppGpp have been yet found in animal cells, but an enzyme capable of hydrolysis of (p)ppGpp, called Mesh1 has been detected in worms, flies, and humans (Sun et al., 2010). The human version of Mesh1 has been found to hydrolyze (p)ppApp and NADPH as well as (p)ppGpp (Ding et al., 2020; Jimmy et al., 2020). Whether NADPH is the *bona fide* substrate of Mesh1 is still a matter of debate, considering that the Drosophila version did not reduce the cellular pools of NADPH when expressed in *Escherichia coli* (Zhu and Dai, 2019).

**Figure 1:**
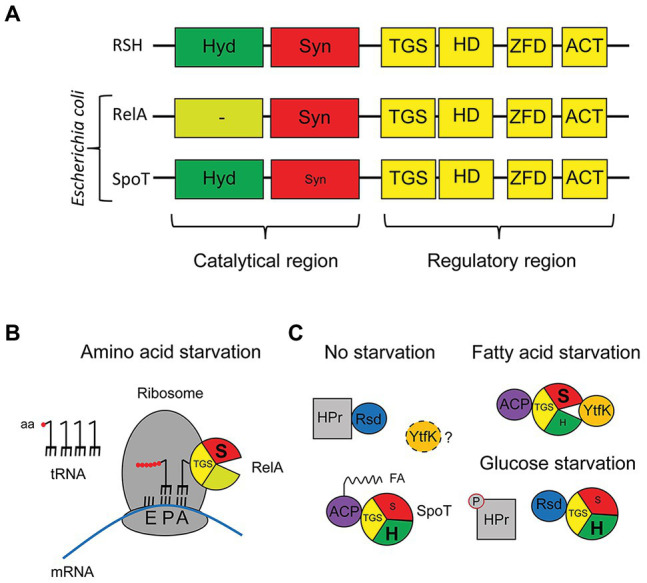
Long RSH enzymes in *Escherichia coli*. **(A)** Cartoon of the domain distribution of a general RSH enzyme as described by Atkinson et al. (2011), as well as for the two RSH enzymes of *E. coli*, RelA and SpoT. **(B)** Schematic representation of the binding of RelA to the ribosome and its activation during amino acid starvation. **(C)** Illustration of the different proteins that bind to SpoT from *E. coli* under non-starvation conditions, fatty acid starvation, or glucose starvation.

Many proteins with (p)ppGpp synthetase and/or hydrolase activity are found among diverse bacteria (Atkinson et al., 2011; Jimmy et al., 2020). Long RelA/SpoT Homologue (RSH) enzymes (Figure 1A), often about 750 residues, contain domains responsible for both activities, hydrolysis and synthesis, followed by regulatory domains. Although all bacteria have bifunctional long RSH enzymes, some can also have monofunctional enzymes (Atkinson et al., 2011; Avilan et al., 2019). To avoid futile cycles of synthesis and hydrolysis, this bifunctional enzyme balances both activities by undergoing conformational changes that activate one activity while inhibiting the other, as shown by structural studies made with the RSH catalytic region of *Streptococcus equisimilis* or *Thermus thermophilus* (Mechold et al., 2002; Hogg et al., 2004; Tamman et al., 2020). Binding of other proteins to the RSH enzyme seems to promote those conformational changes in response to environmental changes (Battesti and Bouveret, 2006; Chen et al., 2014; Lee et al., 2018; Germain et al., 2019; Ronneau et al., 2019; Peterson et al., 2020). Also, RSH enzymes seem to be subject to positive allosteric regulation by their products (Shyp et al., 2012; Kudrin et al., 2018).

In addition, single domain, small alarmone synthetases (SASs), and small alarmone hydrolases (SAHs) are also encountered, sometimes with multiple or additional domains. It has been shown that SAS examples can also synthesize pGpp using GMP as a substrate, with putative physiological roles (Gaca et al., 2015b; Ruwe et al., 2017; Yang et al., 2019; Petchiappan et al., 2020).

After accumulation, (p)ppGpp will change gene expression patterns by binding to RNA polymerase (RNAP) in Gram-negative bacteria or in Gram positives by lowering GTP levels, which is sensed by the transcription factor CodY (reviewed by Gaca et al., 2015a). Alternatively, (p)ppGpp can directly bind to some proteins and alter their synthetic activities (Zhang et al., 2018; Anderson et al., 2019; Wang et al., 2019, 2020).

### Synthesis and Hydrolysis of (p)ppGpp in *Escherichia coli* and Downstream Effects

*Escherichia coli* contain two long RSH enzymes: RelA and SpoT (Figure 1A). While SpoT is a bifunctional enzyme, RelA is a monofunctional enzyme with a non-functional hydrolase domain, making SpoT the only source of hydrolysis (Xiao et al., 1991). Apart from the N-terminal catalytic region of the protein, RelA and SpoT contain a C-terminal regulatory region, displaying highly conserved domains (Figure 1A). Although several C-terminal domains are important for binding to ribosomes or synthesis fine-tuning (Loveland et al., 2016; Takada et al., 2020), the ThrRS, GTPase, SpoT/RelA domain (TGS) domain seems to often be important for controlling the conformational change that these proteins endure to control (p)ppGpp synthesis.

RelA binds to the ribosome by burying its C-terminal region inside the ribosome, just exposing the catalytic region to the cytoplasm. RelA will synthesize (p)ppGpp in response to amino acid starvation (Figure 1B) thorough first sensing cognate binding of uncharged transfer RNA (tRNA) to the ribosomal A-site followed by binding of RelA C-terminal region to the ribosome that leads to fixing the position of the TGS domain such that it can fit the uncharged, but not charged, CCA bases of the 3' end of the tRNA in a pocket (Arenz et al., 2016; Brown et al., 2016; Loveland et al., 2016). The final stabilizing contact between the RelA N-terminus region and Ribosome is viewed as a final lock. While the RelA synthetase domain contacts the tip of the 16S ribosomal RNA (rRNA) spur, the non-functional RelA hydrolase domain will bind near the sarcin-ricin loop of the 23S rRNA (Loveland et al., 2016; Winther et al., 2018). Loveland et al. (2016) note that this model is consistent with a stable tRNA-ribosome idling reaction rather than the extended hopping model that hypothesizes that RelA can dissociate from ribosomes after being activated and remain capable of multiple rounds of (p)ppGpp synthesis after dissociation (Haseltine et al., 1972; Haseltine and Block, 1973; Wendrich et al., 2002). Alternatively, conflicting evidences show that free RelA can stably bind uncharged tRNA and then bind to ribosomes (Winther et al., 2018). The description of the complex mechanisms at play is an extremely active field at the moment but it is not the main topic of this review.

In contrast to RelA, the N-terminal region of SpoT in *E. coli* encodes a strong hydrolase along with a weak synthetase, and the C-terminal region of SpoT that has four domains similar to those in RelA. It is still uncertain if SpoT interacts with ribosomes. So far, two proteins have been found to bind to the TGS domain of SpoT in response to starvation (Figure 1C). They are acyl carrier protein (ACP; fatty acid limitation) and Rsd (glucose limitation). While the acylated form of ACP (in presence of fatty acids) binding to SpoT will tilt the balance against synthesis and toward hydrolysis, the binding of the unacylated form (starvation) will promote synthesis (Battesti and Bouveret, 2006). The binding of Rsd to SpoT will tilt the balance toward hydrolysis, but it will only occur during glucose starvation, when Rsd is released from the phosphorylated form of HPr, a key component of the glucose phosphotransferase system (PTS; Lee et al., 2018).

Other interactions not involving the TGS has been described for SpoT. During phosphate and fatty acid starvation, YtfK binds to the catalytic region and tilts the balance toward synthesis (Germain et al., 2019). Structural studies with the *Thermus thermophilus* RSH protein, mentioned earlier, show that the hydrolysis and synthesis domains are in an open conformation during synthesis but closed during hydrolysis (Tamman et al., 2020). Therefore, considering that YtfK binds to both the hydrolase and synthetase domains (Germain et al., 2019); one could speculate that YtfK could bind between both domains to hold SpoT in the open conformation (Figure 1C). Further studies are required to integrate the various signaling systems together, like a possible synergy between ACP and YtfK to activate SpoT synthetase during fatty acid starvation or a possible competition between ACP and Rsd. Moreover, not much is known about YtfK activation: does it sense the presence of certain nutrients like ACP? Is it sequestered as Rsd? or does its gene expression change under certain conditions? These interactions for SpoT again raise mirror image questions to those raised for RelA. What are the functions of the C-terminal domains beyond TGS if SpoT does not require ribosomal binding for activity? Is it to preserve the weak but constitutive synthetase catalytic activity allowing non-RelA regulation to occur through metabolic signals other than uncharged tRNA? Again, structural studies seem needed for SpoT binding proteins. Another example of existing regulatory intricacies comes from the finding that starvation for either glucose or lipids can deplete amino acid precursors and activate RelA in addition of SpoT (Fernández-Coll and Cashel, 2018; Sinha et al., 2019).

In *E. coli*, as mentioned above, it has been thought for many years that (p)ppGpp exerts its effects mainly by binding to RNAP and changing gene expression by either stimulating or inhibiting transcription of separate sets of genes. Structural studies have shown that (p)ppGpp binds to two different sites in RNAP. Site 1 binding of (p)ppGpp is between the ω and β’ subunits (Mechold et al., 2013). For site 2, (p)ppGpp binds inside the secondary channel of the RNAP, between the β’ subunit and the cofactor DksA (Ross et al., 2016; Molodtsov et al., 2018). Both (p)ppGpp and DksA can act as cofactors for regulation of many genes, but they can also have independent or even opposite effects on gene expression (Magnusson et al., 2007; Aberg et al., 2008, 2009). This co-regulation of genes by (p)ppGpp and DksA is consistent with binding at site 2, but the effects involving site 1 are not so clear. Antagonistic effects have been attributed to other proteins that, like DksA, can also bind to the secondary channel, such as GreA (Aberg et al., 2009), suggesting that the competition of proteins for the secondary channel can change the nature of site 2 (Potrykus et al., 2006; Vinella et al., 2012; Yuzenkova et al., 2012; Zenkin and Yuzenkova, 2015; Fernández-Coll et al., 2018).

## Effects of Basal Levels of (p)ppGpp in *Escherichia Coli*

**Figure 2:**
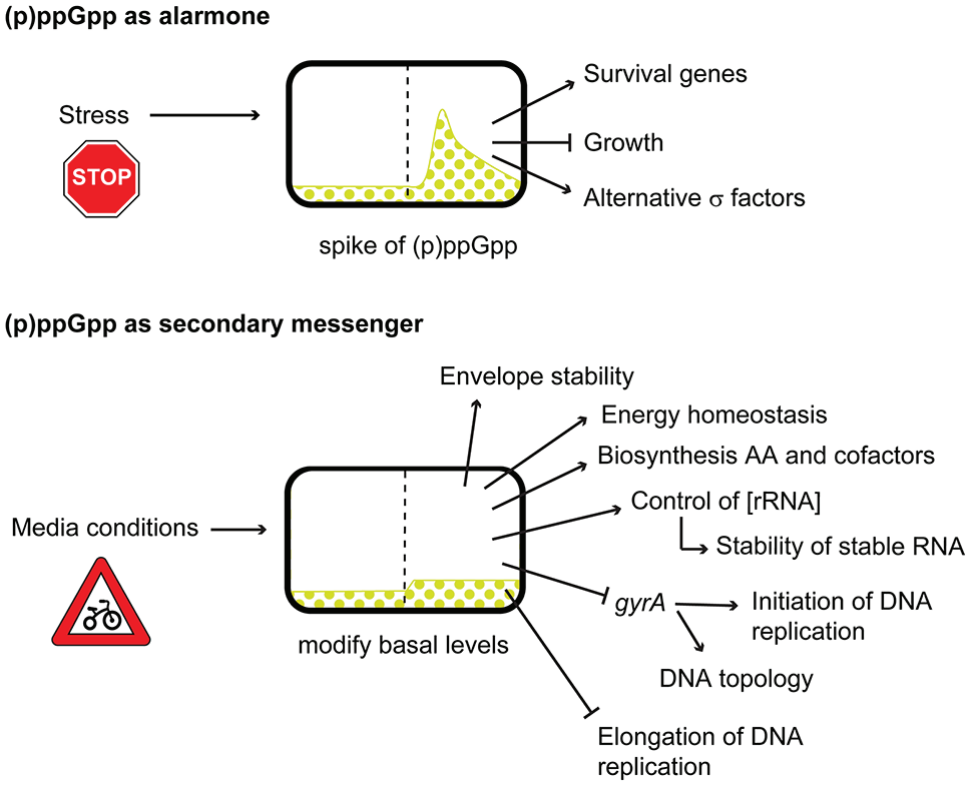
Dual effects of (p)ppGpp acting as alarmone during burst or acting as a secondary messenger during changes in basal levels.

(p)ppGpp seems to have different roles depending on its abundance in the cell. While sudden burst of (p)ppGpp during stress, starvation, or stationary phase, will stop cellular growth, and cells will go into survival mode (alarmone); the low basal levels, during exponential phase, in absence of starvation, (p)ppGpp will meld the external conditions with the bacterial growth, maintaining the homeostasis of cellular components and macromolecules, acting as a secondary messenger (Figure 2). It is because of this duality between basal levels and stressful spikes of (p)ppGpp, and more particularly in the effect of basal levels, that we decided on the focus of this review.

When bacteria grow at constant temperature without starvation, all cellular components are synthesized exponentially. The subtle intricacies of the balanced exponential growth have long been fascinating (Neidhardt, 1999). As originally defined by Schaechter M., Maaloe O., and Kjeldgaard N.O. in 1958, balanced growth rates were determined not by limiting nutrient abundance but by the ability of the cell to use different nutrients in excess. Using those conditions, an inverse correlation is found between the levels of (p)ppGpp and growth rate, where higher levels of (p)ppGpp correlate with lower growth rates (Imholz et al., 2020).

The exponential phase of growth transits into stationary phase, which is associated with regulation often directly or indirectly due to elevated (p)ppGpp as well as a myriad of additional mechanisms that include recruitment of alternative sigma factors and ribosome hibernation (not reviewed here). A major contribution comes from preventing RpoS sigma factor proteolysis, stabilized during stationary phase, or nutritional stress due to (p)ppGpp induction of the sRNA IraP (Bougdour and Gottesman, 2007; Gottesman, 2019). Other alternative sigma factors are also activated by (p)ppGpp (Österberg et al., 2011). AT rich and GC rich discriminator sequences at the promoter regions also determine whether (p)ppGpp stimulates or inhibits transcription, respectively (Travers, 1980; Winkelman et al., 2016; Sanchez-Vazquez et al., 2019).

### Basal (p)ppGpp Levels and Cellular Component Homeostasis

Inhibition of key enzymes from a dozen of cellular processes has been assessed using CRISPRi in *E. coli* (Roghanian et al., 2019), in either a WT strain or in a *relA*- *spoT*- strain (referred as ppGpp^0^). While WT cells stop growing after inhibition of the different processes with CRISPRi, the ppGpp^0^ cells grow uncontrollably until they lyse, just like a car without breaks going downhill. It was found that CRISPRi inhibition of the biosynthesis of LPS (repressing *lptA* or *lpxA*) or ATP (repressing *adk*) increases the (p)ppGpp levels in wild type cells.

During exponential growth in LB and early stationary phase, the levels of ATP in wild type remain constant, in later stationary phase (more than 20 h) energy charge decreases (Chapman et al., 1971). In ppGpp^0^, the levels of ATP are lower in exponential phase and early stationary phase compared to WT strains; this again suggests basal levels of (p)ppGpp are important for cellular energy homeostasis in *E. coli* by coordinating adenosine ribonucleotide synthesis with macromolecular production (Roghanian et al., 2019). That said, in wild type cells, the cellular levels of ATP do not seem to correlate with various exponential growth rates (Schneider and Gourse, 2004).

Basal levels of (p)ppGpp seem to be important for the stability of the cellular envelope. A mutant of the RNAP β’ subunit (RpoC H419), that seems to have impaired binding of (p)ppGpp to site-1, shows an increase in the average lipid tail length, that results in changes in the cell membrane properties (Chen et al., 2020). A study on the effects of (p)ppGpp into T4 phage infection seems to reach, indirectly, similar conclusions. T4 phage plaques in *E. coli* show an increase on plaque size in absence of either (p)ppGpp or DksA (Patterson-West et al., 2018). Although DksA affected T4 gene expression, (p)ppGpp had almost no effect on the phage gene expression, suggesting that its effects on plaque size are related to effects in the host rather than the phage, which could be attributed to an increased membrane fragility in absence of (p)ppGpp (Patterson-West et al., 2018).

A useful phenotype of (p)ppGpp-deficiency is an inability to grow in minimal media without a set of eight amino acids (Xiao et al., 1991). These amino acids (DEILVFHST) are known as the Σ set (Potrykus et al., 2011). Interestingly, a few of the same amino acids are also required for ppGpp^0^ in *Bacillus subtilis* (Kriel et al., 2014). Transcriptomic studies performed during amino acid starvation, show that (p)ppGpp is essential to activate several biosynthetic pathways to produce the amino acids of the Σ set (Traxler et al., 2008). At the same time, studies with ppGpp^0^ synthetic lethal mutants reveal that (p)ppGpp activates several key components to produce D-erythrose-4-phosphate, the precursor of aromatic amino acids and vitamins (Harinarayanan et al., 2008). Apparently, basal levels of (p)ppGpp are important for uptake of iron (Vinella et al., 2005). This suggests that basal levels of (p)ppGpp are essential for biosynthesis of a growing list of metabolic intermediates, amino acids, and cofactors.

Incremental lowering of already low basal levels of (p)ppGpp by expression of the metazoan SAH Mesh1 in *E. coli* has been reported to slow growth and inferred to extend the inverse relation of ribosomal content and (p)ppGpp (Zhu and Dai, 2019). A direct relation of certain metabolic enzymes with (p)ppGpp, is found suggesting to the authors that (p)ppGpp basal levels are important to keep optimal allocation of resources during balanced exponential growth in *E. coli*. This conclusion rests on the assumption that Mesh1 activity is limited solely to (p)ppGpp hydrolysis. Traces of (p)ppApp have been detected by TLC in *E. coli* (Sobala et al., 2019), therefore, until the full range of Mesh1 substrates is known within *E. coli*, conclusions as to the mechanism of growth inhibition by Mesh1 are qualified.

### Does Instability of tRNA and rRNA Depend on (p)ppGpp?

For more than a half century, with a few exceptions, it has been assumed that tRNA and rRNA are stable species, and regulation of their cellular abundance by starvation or by growth rate was exerted solely at the level of transcription. During the past 2 decades, studies with *E. coli* have focused on the mechanisms involving (p)ppGpp that could account for the many of the inhibitory and stimulatory regulatory effects on transcription.

The first systematic glimpses of rRNA turnover came from studies by Murray Deutscher’s laboratory that were aimed at understanding the differences they found for ribonuclease sites for degradation of 30 and 50S ribosomal subunits during exponential growth *versus* more sites found for stationary phase and glucose-starved culture (Sulthana et al., 2016). A recent review of relevant enzymes in *E. coli* and *B. subtilis* has appeared (Bechhofer and Deutscher, 2019). For tRNA, a convincing demonstration of destabilizing effects on tRNA during abrupt starvation by filtration and resuspension for each of several individual amino acids appeared in a paper by (Svenningsen et al., 2016). Quantitating effects were normalized to a unique internal standard isolated from *Sulfolobus sulfutaricus*. Although the authors report general decay kinetics for a *relA* mutant to be similar to wild type, degradation attenuation can be observed in absence for *relA*. One example can be observed for HisR, that decreases down to a 30% after 60 min of histidine or leucine starvation in the wild type cells, but in a *relA* mutant up to a 50% remains after histidine starvation, and up to a 70% after leucine starvation. Despite of that, it was argued to indicate that degradation is independent of (p)ppGpp, since (p)ppGpp levels are reduced in an amino acid starved relaxed strain. Instead, a tRNA demand-based model has been proposed in which tRNA not engaged in protein synthesis is vulnerable to degradation; cells in slow exponential growth are stated to have more tRNAs per ribosome than during fast growth with more active ribosomes. It is argued that increased EF-Tu content during slow growth binds the free tRNA to protect it from degradation (Sørensen et al., 2018).

Unstable rRNA has been also reported, using a similar normalization method than with tRNA studies. Fessler et al. (2020) looked at the kinetics of different filter-resuspend starvation regimens, showing that while glucose starvation produces minimal instability of rRNA, isoleucine and phosphate starvation reduced ribosome content in a similar manner than previosly observed by tRNA (Svenningsen et al., 2016). Again, the authors concluded that although (p)ppGpp may contribute, it is not essential for the degradation of rRNA, instead, inactive ribosomes are vulnerable to degradation. Therefore, it is possible that ppGpp affects rRNA stability indirectly by affecting translation. (p)ppGpp can bind to several GTPases involved in translation or ribosome biogenesis, including IF2, inhibiting their activity (Zhang et al., 2018).The end result would be a decrease on the number of active ribosomes, now susceptible of degradation. Interestingly, the inhibitory effects of (p)ppGpp over IF2 activity may depend on the mRNA being translated (Vinogradovaid et al., 2020).

As often noted here, growing *E. coli* in media with excess nutrients without starvation classically show a direct correlation between growth rates and RNA/DNA and RNA/protein ratios. This correlation is broken in the total absence of (p)ppGpp; instead, high ratios characteristic of fast growth persists even during slow growth (Potrykus et al., 2011). It can be shown that this aberrant behavior is due to (p)ppGpp and not to stress using constitutive elevated (p)ppGpp at different levels displayed by *spoT* hydrolase (*relA*^−^) mutants that grow slowly in rich media as if it were poor (Sarubbi et al., 1988). The observed low ratios characteristic of slow growth for these mutants is consistent with the notion that (p)ppGpp is necessary and sufficient for slowing growth rates (Potrykus et al., 2011). Moreover, RNAP suppressor mutants isolated from ppGpp^0^ strains that grow as prototrophs in minimal media lacking amino acids and grow slowly even rich media (Murphy and Cashel, 2003). This indicates that slow growth of RNAP mutants is a phenocopy of (p)ppGpp levels that can be exerted through transcriptional effects. Sucrose gradient comparisons of ribosomal content for slow and fast growing (p)ppGpp-deficient cells reveal normal ribosomal profiles that suggest they might be functional and more abundant than needed at slow growth rates, and yet the overall rate of translation is reduced compared to WT cells (Potrykus et al., 2011).

Differential effects on kinetics of *lacZ* transcripts have been observed on transcription and translation rates depending on overt starvation for carbon and nitrogen (Iyer et al., 2018) or steady-state starvation with chemostats for carbon, nitrogen, or phosphate source on the media (Li et al., 2018; see commentary by Potrykus and Cashel, 2018). There is a very good chance that RNA sequencing experiments just mentioned with slow growing ppGpp^0^ cells will also result in high rates of tRNA and rRNA turnover. For completeness it seems worthwhile to verify this expectation by measuring the extent of instability for tRNA and rRNA in exponentially growing ppGpp^0^ strains, because of unforeseen properties of *relA^−^ spoT^−^* strains often not observed when using *relA^−^ spoT^+^* strains.

### Basal (p)ppGpp Levels and DNA

As previously described, during exponential growth, the amount of DNA and the number of replication origins per cell correlate with the growth rate (Churchward et al., 1981). During rapid growth in rich media, the time required to complete chromosomal replication is longer than the time for cell division, therefore, additional rounds of replication are necessarily initiated at the *ori* region before the previous round is completed (Cooper and Helmstetter, 1968). When determining the initiation of DNA replication by *ori*/*ter* ratios, cells growing in rich media show higher ratios of *ori/ter* than cells growing in poor media. However, in absence of (p)ppGpp, initiation of DNA replication becomes independent of the growth rate, showing high constant initiation rates (*ori*/*ter*) despite changing the growth rate by varying media composition and nutrient availability (Fernández-Coll et al., 2020). When the basal levels of (p)ppGpp are gradually increased without stress due to mutations on the SpoT hydrolase, as observed with RNA/DNA and RNA/protein (Potrykus et al., 2011), a proportional decrease on the growth rate and decrease on the *ori/ter* ratios were observed, suggesting that (p)ppGpp is also necessary and sufficient to control DNA replication initiation (Fernández-Coll et al., 2020).

The initial step of chromosomal DNA replication involves the ATP-dependent oligomerization of DnaA to *oriC*, followed by the unwinding of the DNA, loading of the replisome. While (p)ppGpp controls the expression of *dnaA* in *E. coli* (Chiaramello and Zyskind, 1990; Sanchez-Vazquez et al., 2019), the effect on DNA initiation seems to correlate with expression changes of DNA gyrase produced by (p)ppGpp that would change the local supercoiling surrounding the origin (Fernández-Coll et al., 2020). Also, these effects over DNA gyrase will promote changes on the global DNA topology (Travers and Muskhelishvili, 2015), and it will be interesting to determine which effects associated with (p)ppGpp require changes in the supercoiling state of the chromosome. As have been observed in *B. subtilis* and *S. aureus*, (p)ppGpp can also bind to the DNA primase DnaG and slow down elongation of DNA replication in *E. coli* (Wang et al., 2007; Maciag et al., 2010; Rymer et al., 2012; Maciąg-Dorszyńska et al., 2013).

## Effects of Gradual Accumulation of (p)ppGpp Vs. Sudden Bursts

The differential effects of basal levels of (p)ppGpp compared to high level bursts during stressful situations can be attributed to concentration and accumulation speed. Apart from tRNA and rRNA instability, other studies have revealed that differences between gradual accumulation of (p)ppGpp compared to a sudden burst during isoleucine starvation in *E. coli* K12 strains. In absence of isoleucine, the presence of valine will inhibit *E. coli* K12 strains growth (Leavitt and Umbarger, 1962) due to inhibition of isoleucine biosynthesis (Lawther et al., 1981). Addition of valine will abruptly increase the levels of (p)ppGpp that equal GTP within 5 min after induction (Cashel and Gallant, 1969; Fernández-Coll and Cashel, 2019). This feature was used by Traxler et al. (2011) to progressively induce high levels of (p)ppGpp by growing *E. coli* K12 in media containing all amino acids in excess and almost limiting amounts of isoleucine that are exhausted during growth to the point where valine progressively induces starvation for isoleucine. As the levels of (p)ppGpp gradually increase, the first activation detected is a set of Lrp-dependent genes. Further, (p)ppGpp elevation is then followed by activation of RpoS and downstream by the set of genes under σS control. These results suggested that under a starvation stress situation, cells will first try to relocate resources to restore the normal growth, but as the amounts of (p)ppGpp keep rising, they will go into survival mode. Lrp-dependent regulon gene expression was not seen in the RNA sequencing studies during abrupt starvation of isoleucine by adding valine as noted by Gummesson et al. (2020). Moreover, abrupt starvation provokes unique gene regulation. such as *crp*, that increases at the 5 min peak of high (p)ppGpp levels during abrupt starvation, which is followed by lowering of gene expression to pre-induction levels or even lower (Gummesson et al., 2020).

Another study showing the effects of a (p)ppGpp on *E. coli* K12 strain expression pattern over time was recently published (Sanchez-Vazquez et al., 2019). In this study, the authors grow MG1655, together with several alleles that abolish the two binding sites of (p)ppGpp in RNAP. In MOPS with all the amino acids and IPTG induced RelA, a burst of (p)ppGpp occurs without starvation. When Gummesson et al. (2020) compared their results with those of Sanchez-Vazquez et al. (2019); they found big differences on the expression levels of genes involved in amino acid biosynthesis that they attributed to the media conditions. This suggests that, although in both experiments a burst of (p)ppGpp is being produced, the media conditions will affect the outcome.

It is important to note that the RNAP mutant strain lacking the both binding sites of (p)ppGpp (used in Sanchez-Vazquez et al., 2019), should behave as a (p)ppGpp-deficient strain and not be able to grow in minimal media (Xiao et al., 1991); it grows slowly on minimal medium (Ross et al., 2016). One possible explanation for the slow growth without amino acids is that while (p)ppGpp might not bind to the double site mutant RNAP, it still could have unappreciated functions at the metabolic level. Another possibility is that the RNAP mutant might confer similar conformational changes to those observed in ppGpp^0^ spontaneous mutants that grow on minimal media without amino acids (Murphy and Cashel, 2003).

## Is What is True for *Escherichia Coli*, True for Elephants and Other Bacteria?

The duality between basal levels of (p)ppGpp and stressful peaks has been observed in other organisms. When the genes affected by (p)ppGpp during exponential phase in *Rhizobium etli* were compared to those affected during stationary phase (Vercruysse et al., 2011), only 25% were found to be shared between both phases and of those, only half were similarly positive or negative controlled. This study clearly emphasizes the differential effects of basal levels of (p)ppGpp during exponential phase compared to a peak of (p)ppGpp during stationary phase.

In the Gram-negative bacteria *Caulobacter crescentus*, its complex cell cycle is controlled by (p)ppGpp, that affects gene expression and degradation of the main regulatory proteins DnaA and CtrA with antagonistic activities. DnaA activates initiation of DNA replication, and CtrA blocks it (Lesley and Shapiro, 2008; Boutte et al., 2012; Gonzalez and Collier, 2014; Stott et al., 2015).

As previously discussed, in Gram-positive bacteria (p)ppGpp will affect gene expression by changing the levels of GTP, sensed by CodY. Therefore, it is essential for Gram-positive to keep the homeostasis of GTP. In *B. subtilis*, increased levels of (p)ppGpp block the biosynthetic enzymes HprT and GmK that are essential for the production of GTP (Kriel et al., 2012; Anderson et al., 2019). The enzyme HprT, as a dimer, will synthesize GMP, but the binding of (p)ppGpp leads to formation of tetramers that are inactive (Anderson et al., 2019). Similar effects have been observed for GmK. Phylogenetical studies show that this is conserved among Gram-positive bacteria, but in Gram-negative bacteria, these enzymes are insensitive to (p)ppGpp (Anderson et al., 2019). In this work, the authors substituted *B. subtilis* HprT and GmK enzymes for the ones in *E. coli* (p)ppGpp insensitive enzymes that show an increase of GTP levels up to four times without stress. This is taken by the authors to underscore the conclusion that basal levels are essential in Gram-positive to maintain GTP homeostasis. Apart from its regulatory effects, maintaining low GTP levels is essential because high levels of GTP seem to be toxic for *B. subtilis* cells (Kriel et al., 2012). *Bacillus* contains a long bifunctional RSH enzyme (Rel) mediating the response to nutritional starvation (Wendrich and Marahiel, 1997; Pulschen et al., 2017; Takada et al., 2020), as well as two SASs (RelP and RelQ). It has been observed that while RelP is always active, RelQ requires pppGpp to be active, suggesting that RelQ will act as amplifier of the response of Rel during stress situation (Steinchen et al., 2018). Moreover, RelQ seems to be inhibited by the binding of certain single stranded RNA (Beljantseva et al., 2017). In contrast, RelP seems to be responsible for the basal levels of (p)ppGpp (Ababneh and Herman, 2015). In absence of Rel hydrolase activity there is an increase on the basal levels due to the SAS, producing a change from chained cells to unchained motile cells; thus, minor increases of (p)ppGpp basal levels will promote important cell changes (Ababneh and Herman, 2015). There is a report where the amount of uncharged tRNA was determined not by amino acid starvation, but by underproduction of aminoacyl tRNA synthetases (aaRS) in rich media conditions. It shows that (p)ppGpp enhances growth when there is insufficient aaRS activity in the absence of external starvation in *B. subtilis* (Parker et al., 2020). Interestingly, as they reduce the fitness by underproducing aaRS, the amount of ribosomal proteins decreases in WT cells, but they keep constantly high in absence of (p)ppGpp, reminiscent of the observed behavior of ribosomes in *E. coli* (Potrykus et al., 2011). In Parker et al., (2020), the optimal growth happens when the amount of tRNA charging is not maximized and that (p)ppGpp is key to maintain the protein stoichiometry for the translation apparatus. Studies performed in tRNA maturation in *B. subtilis* (Trinquier et al., 2019) show that accumulation of immature tRNA triggers synthesis of (p)ppGpp that will interfere in the maturation of the 16S rRNA. Together with the data from Parker et al. (2020) seem to suggest that (p)ppGpp accommodates the number of ribosomes to the amount of functional tRNA. In contrast, a spike of (p)ppGpp during heat shock, seems to protect 16S rRNA from degradation (Schäfer et al., 2020), suggesting that the effects of basal levels of (p)ppGpp may have even opposite roles than spikes of (p)ppGpp suggesting that stress-dependent factors may be required.

A study made in the cyanobacterium *Synechococcus elongatus* has shown differential roles between basal levels of (p)ppGpp and spikes during stress (Puszynska and O’Shea, 2017). During growth with constant light (no stress), the basal levels of (p)ppGpp in *S. elongatus* are responsible for the control of protein levels and bacterial size. During the transition from light to dark, a spike of (p)ppGpp is produced inhibiting up to a 90% of the transcripts (Hood et al., 2016; Puszynska and O’Shea, 2017). This spike of (p)ppGpp increases the expression of *hfp*, a factor responsible for reducing the activity of ribosomes by promoting their dimerization, that will also decrease the levels of photosynthetic pigments and will stop growth (Hood et al., 2016).

## Basal Levels of (p)ppGpp and Pathogenicity

Levels of (p)ppGpp have been shown to be important for virulence or survival inside the host of different bacteria, like *Salmonella enterica* serovar Typhimurium that in absence of (p)ppGpp was found to be highly attenuated *in vivo* and non-invasive *in vitro* (Pizarro-Cerdá and Tedin, 2004). In this case, the levels of (p)ppGpp will spike after invasion due to the lack of nutrients or due to oxidative stress and acidic pH inside macrophages (Fitzsimmons et al., 2018, 2020). Even commensal *E. coli* expresses factors essential for biofilm and colonization of surfaces such as fimbria, flagella, or antigen 43, just after the peak of (p)ppGpp during stationary phase (Aberg et al., 2008, 2009; Cabrer-Panes et al., 2020).

However, a few examples exist where the basal levels of (p)ppGpp are also essential for virulence. *Enterococcus faecalis* is a Gram-positive bacterium responsible for approximately 30% of bacterial infectious endocarditis cases in the world (Chirouze et al., 2013). It contains a long bifunctional RSH enzyme (Rel) responsible for increasing the levels of (p)ppGpp in response to stress (nutritional and physical) and the SAS RelQ (Abranches et al., 2009). Basal levels of (p)ppGpp were found to be responsible for controlling energy production and for maintaining the GTP homeostasis (Gaca et al., 2013), but also are essential for heart valve colonization and endothelial cells invasion of human coronary arteries during infectious endocarditis (Colomer-Winter et al., 2018). Similar observations were made in *Mycobacterium tuberculosis*, where the basal levels of (p)ppGpp were modified using mutants in the long RSH enzyme Rel. Too low or too high levels of (p)ppGpp were proven to be lethal for *M. tuberculosis* during acute and chronic infections in mice, which seems to be a Goldilocks effect: not too little, not too much, and just right (Weiss and Stallings, 2013).

Considering the effects of (p)ppGpp on virulence factors and its absence in metazoan cells, several groups have pointed to the synthetases of (p)ppGpp as a target to develop new antibiotics. According to the CDC’s Antibiotic Resistance Threats 2019 Report, each year in the United States, at least 2.8 million people are infected with antibiotic-resistant bacteria and more than 35,000 people die as a result. This number is estimated to escalate, someday rendering current antibiotic treatment completely obsolete.

Serious attention has been given to new antibiotic development based on microbial (p)ppGpp and its absence in eukaryotes. Exploitable possibilities are many, since so far, the ppGpp^0^ state is generally associated with reduction of pathogenicity, basal levels with pathogenicity, and high levels with cessation of bacterial growth. At this point, there are no easy answers. Attempts have been made to produce (p)ppGpp analogs that interfere with the synthesis of (p)ppGpp (Wexselblatt et al., 2012; Beljantseva et al., 2017). A minor problem of that strategy is that the high polarity of (p)ppGpp and analogs interferes with their permeability, but this can be bypassed by chemically cloaking the nucleotide analogs with nonpolar tags, making them permeable. A troublesome problem is that strategies that block synthesis of (p)ppGpp even by inactivating multiple synthetases would be rendered impractical because of spontaneous RNAP suppressor mutants in (p)ppGpp-deficient strains, able to mimic the presence of (p)ppGpp (Murphy and Cashel, 2003; Kriel et al., 2012). Moreover, compounds that inhibit the long RSH enzymes are found, so far, to be inefficient inhibitors of SASs from *E. faecalis* (Gaca et al., 2015b; Beljantseva et al., 2017).

Perhaps, an alternative strategy would be to target the hydrolase domain of the RSH enzymes. As often mentioned above, (p)ppGpp inhibits bacterial growth. Analogs that give sufficient increases of intracellular of (p)ppGpp should severely slow growth, which could be interesting antibacterial target. Learning from different SpoT hydrolase mutants, such as SpoT 202 or 203 (Sarubbi et al., 1988), or the known binding of proteins to long RSH enzymes would help developing compounds able to tilt the balance through synthesis or blocking hydrolysis. This is also a slippery slope because increased (p)ppGpp often has been associated with antibiotic persistence (Svenningsen et al., 2019), although the mechanisms are still unclear. Development of drugs that give constitutive hydrolysis is another option. This would minimize interference by laterally transmitted synthetases as one mechanism of achieving drug resistance (Jimmy et al., 2020).

## Concluding Remarks

In this review, we address effects of basal levels of (p)ppGpp on bacteria physiology with focus on *E. coli*, what we know best. However, when we try to expand it to organisms less familiar to us or when we try to generalize using model organisms, it is good to remember the variety of organisms and how they have adapted differently to synthesize (p)ppGpp and respond to its accumulation.

The duality of (p)ppGpp between “alarmone” during stress and secondary messenger during exponential growth seems to depend not only on the amount of (p)ppGpp, but also on the rate of its accumulation. Most studies use a burst of (p)ppGpp or excruciating starvation conditions to try to estimate physiological effects of (p)ppGpp. Some of those methods will highly increase the levels of (p)ppGpp even higher than direct starvation methods. For example, producing starvation with serine hydroxamate (SHX) or the overexpression of the catalytical region of a RSH enzyme, is useful tools to produce a burst of (p)ppGpp, but lack the feedback control mechanisms that starvation or stress may have. By pushing cells so far, one may end up making conclusions from close-to-death cells.

An alternative method is to use (p)ppGpp-deficient cells, it is far from perfect either. It will reveal a need for (p)ppGpp but not the specific mechanistic target. As previously discussed, these strains will not grow in minimal media without certain amino acids and will require a supplement of iron in the media, rendering some experiments impossible. The addition of the set of eight amino acids essential for *E. coli* provides a way to grow (p)ppGpp-deficient cells in poor media, but then we need to look for the appearance of suppressor mutants that may mask the results.

Most of basal levels of (p)ppGpp are described during balanced growth, where growth rate classically depends on the ability of bacteria to use certain nutrients, instead of their availability in the media. However, in several studies, they use chemostat cultures to achieve “balanced growth.” We should say that from our perspective, chemostat steady state growth limits nutrients available and therefore systematically varies the intensity of starvation, which is likely to be very different from subtle cellular adjustments needed to optimize the efficiency of metabolizing otherwise poorly utilized nutrients.

Future work will try to navigate through these difficulties. It will probably involve designing strategies that will help distinguish between brute force and fine-tuning effects on bacterial physiology.

## Author Contributions

LF-C and MC conceived, drafted, and revised the manuscript. Both authors have read and approved the final version of this manuscript.

### Conflict of Interest

The authors declare that the research was conducted in the absence of any commercial or financial relationships that could be construed as a potential conflict of interest.
